# Self-Perception of Periodontal Health and Associated Factors: A Cross-Sectional Population-Based Study

**DOI:** 10.3390/ijerph17082758

**Published:** 2020-04-16

**Authors:** Federica Romano, Stefano Perotto, Laura Bianco, Francesca Parducci, Giulia Maria Mariani, Mario Aimetti

**Affiliations:** 1 Department of Surgical Sciences, C.I.R. Dental School, Periodontology Section, University of Turin, 10100 Turin, Italy; federica.romano@unito.it (F.R.); laura.lb.bianco@gmail.com (L.B.); francesca.parducci@yahoo.com (F.P.); giuliamaria.mariani@unito.it (G.M.M.); 2 Postgraduate Program in Periodontology, Department of Surgical Sciences, University of Turin, 10100 Turin, Italy; stefanoperotto@libero.it

**Keywords:** cross-sectional study, gingival bleeding, self-perception, tooth mobility, periodontitis, oral malodor

## Abstract

The aim of this cross-sectional study was to explore sociodemographic, behavioral, and clinical factors associated with self-awareness of periodontal health. Data were collected from a representative sample of 736 adults (25–75 years old) in a city of Northern Italy who self-assessed gingival bleeding, oral malodor, and tooth mobility in a questionnaire and who underwent clinical periodontal examination and organoleptic evaluation. Approximately 50% of the subjects were aware of their actual gingival health status and oral odor. The logistic regression analysis revealed that females presented higher odds of correctly perceiving their gingival conditions and mouth odor, while those who were older and smokers had a greater probability of being less objective in reporting them. Tooth type and position in the dental arches were positively associated with self-perception of tooth mobility. These findings reflected a low level of self-awareness that may influence oral care-seeking behavior. Subjects may be unconcerned about their periodontal health condition or lack enough knowledge to be aware of it. This points to the need for planning strategies to improve education and knowledge about periodontal health, which, by enhancing self-perception of periodontal symptoms, could help everyone to seek treatment in the initial stage of the disease.

## 1. Introduction

Periodontal disease is a widely prevalent oral health problem. It is estimated that almost half of subjects over 30 years old are affected by periodontitis in the United States [1,2], and even higher prevalence has been reported in Europe [3]. The key presentations of periodontitis in the early stages are gingival bleeding, recession of the gingival margin, and halitosis and in advanced disease, hypermobility, migration, and tooth loss resulting in impaired oral function, esthetics, and quality of life [4]. 

A mandatory precondition for periodontal treatment is the patient seeking consultation about a sign or symptom recognized as abnormal. However, periodontitis is a silent disease in which pathological changes take a long time before pain, discomfort, and functional disability occur [5]. Therefore, people often underestimate the presence and severity of periodontal disease and seek treatment when advanced attachment loss has already occurred [6,7]. 

The perception of “health” or “disease” is not only related to the severity of signs and symptoms, but it is a reflection of multidimensional sociodemographic and cultural backgrounds at both the population and individual level [8]. In larger populations, a combination of demographic measures and self-reported oral health questions (self-perceived assessment) has demonstrated promising validity in predicting periodontitis for planning public health programs, especially when clinical assessment is unattainable [9,10,11,12]. In individuals, clinical and self-reported measures of periodontal disease have been reported to demonstrate significant disparity [13,14]. 

A recent contribution provided evidence of low sensitivity (disease perception), but high specificity (health perception) values for self-reported bleeding gums [13,14] and low to moderate sensitivity and specificity for oral malodor [15,16,17,18], while little is known about self-perceived tooth mobility [15,16,19,20]. In contrast, people demonstrated the ability to refer to their own dental history, number of remaining teeth, previous experience of restorations, presence of prostheses, and screening of urgent dental care [6,9,21,22,23,24].

Several studies have shown that self-perception of oral health varies among social groups and age cohorts [13,25,26], although these findings have not been corroborated in other studies [27,28]. Most of the available information is derived from older participants, especially community dwelling or institutionalized individuals [29,30,31], while representative samples of adult populations were seldom involved, and none of them assessed predictors of gingival health and oral odor self-awareness [5,10,32]. Self-awareness of periodontal health status influences oral health-seeking behavior and is related to the utilization of dental services for early detection and prevention of periodontal disease.

Therefore, the aim of the present study was to explore sociodemographic, behavioral, and clinical factors associated with self-awareness of periodontal health in terms of gingival bleeding, oral malodor, and tooth mobility in a representative adult population in Northern Italy. 

## 2. Materials and Methods

The dataset from this study came from the data of a cross-sectional population-based epidemiological survey examining the prevalence of periodontitis and halitosis [3,33]. The survey was approved by the Institutional Research Ethics Committee (Protocol No. 0082388), and informed consent was obtained by each survey participant. Data were collected through administration of a structured questionnaire and clinical oral examination between 1 December 2009 and 31 July 2010 by the Section of Periodontology, C.I.R. Dental School, Department of Surgical Sciences, University of Turin (Italy). This study was reported in strict compliance with the STROBE statements.

### 2.1. Study Population

In Italy, all residents are assigned a public general practitioner (GP) and enrolled in the Health Regional Registries. The survey used a two-stage probability sampling method from the Health Regional Register of Piedmont to collect a representative sample of the adult population of Turin, an industrialized city in the northwest of Italy. In the first stage, the units of selection were the GPs, stratified by the four health care districts of Turin to ensure geographic and socioeconomic coverage. The second stage consisted of the random selection of the subjects cared for by each GP. A total of 736 dentate subjects, aged 20 to 75 years old, agreed to take part in the study. A more extensive description of the sampling design was previously published [3].

### 2.2. Structured Questionnaire

Prior to the clinical examination, all study participants were asked to complete a self-administered questionnaire on their self-perception of periodontal health. The three self-report questions all had binary (yes/no) response categories and are listed here with reference abbreviations in parentheses: (1) Do your gums bleed after you brush your teeth (“gum bleeding”)? (2) Do you think you presently suffer from bad breath (“oral malodor”)? (3) Do you have any loose teeth (“loose teeth”)?

The questionnaire also yielded information about individual socio-demographic factors (e.g., age, gender, ethnicity, education level), lifestyle factors (e.g., smoking status), oral health-related behavioral factors (e.g., brushing frequency; daily use of interdental floss; use of tongue scrapers and mouth rinse; frequency of professional scaling), and medical history. 

### 2.3. Clinical Examination

One experienced and calibrated clinician (S.P.) performed the clinical examination of all participants in the GP’s medical offices. The full examination protocol has been described in detail elsewhere [3]. No information about questionnaire responses was provided to the examiner. 

Clinical diagnosis of oral malodor was based on the organoleptic test (OLT) using the 0–5 Rosenberg point scale [34]. Subjects closed their mouth for 3 min and exhaled the air from the mouth through a paper tube at a distance of about 10 cm from the examiner. Subjects were diagnosed as having clinical oral malodor when their OLT score was 2 or greater [35].

The periodontal examination included assessment of all teeth, excluding third molars, for the presence/absence of bacterial plaque, presence/absence of bleeding on probing, probing depth, gingival recession, and clinical attachment level on six sites per tooth using a manual periodontal probe with 1 mm markings (PCPUNC15, Hu-Friedy^®^, Chicago, IL, USA). Gingival inflammation was expressed as the percentage of bleeding sites of the total number of sites in the dentition (full-mouth bleeding score (FMBS)). Tooth mobility was assessed using a four-grade system (0, I, II, III) [36]. No dental radiographs were made.

Periodontal status was established using the case definitions for severe and moderate periodontitis agreed upon by the Centers for Disease Control and Prevention (CDC)/American Academy of Periodontology (AAP) working groups [37,38]. The classification of no/mild periodontitis was assigned to cases that did not qualify as having severe or moderate periodontitis.

### 2.4. Outcome Measures

The self-awareness of gingival bleeding and oral malodor and the self-perception of tooth mobility formed the outcome variables. The self-awareness of gingival bleeding was calculated by comparing self-reported gingival bleeding with FMBS values. Based on the current literature, the cut-off point for gingival inflammation was set at 10% of FMBS [39]. Thus, subjects with FMBS < 10% who did not self-perceive gum bleeding, as well as subjects with FMBS ≥ 10% who self-perceived gum bleeding were considered aware of their gingival health status. Regarding bad breath, OLT scores were dichotomized into no halitosis (0–1) and clinically detected halitosis (2–5). Subjects with an OLT score of 0–1 who did not self-perceive bad breath odor, as well as subjects with an OLT score of 2–5 who self-perceived oral malodor were considered aware of their oral odor. 

In contrast with the previous outcomes, it was not possible to evaluate the concordance between self-perceived and clinically diagnosed tooth mobility because of the lack of a clinical index incorporating both the presence and severity of tooth mobility on a dentition-wide basis. Thus, self-perceived tooth mobility was considered in this study.

### 2.5. Data Analysis

Data were analyzed using statistical software (SPSS, Version 24, Chicago, IL, USA). Associations of the categorical background variables with outcome variables were examined using the chi-squared test. 

Multiple logistic regression analysis was used to model the relationship among self-awareness of gingival bleeding (yes or no), self-awareness of oral malodor (yes or no), self-perceived tooth mobility (yes or no), and various explanatory variables. Purposeful selection of statistically (*p*-values ≤ 0.2 in the bivariate analyses) and clinically relevant variables was conducted. Explanatory variables entered in the models were age (categorized into 3 groups: <40, 40–59, 60–75 years), gender, education level (categorized into 3 levels: low or primary and secondary school level, intermediate or high school diploma, and high or educational attainment beyond the high school level), smoking status (categorized into 3 levels: non-smoker, light smoker (≤10 cigarettes/day), heavy smoker (>10 cigarettes/day)), FMBS (categorized into 4 levels: 0–29%, 30–49%, 50–75%, >75%), toothbrushing frequency (categorized into 3 levels: ≤once/day, twice/day, ≥three times/day), professional scaling frequency (categorized into 4 levels: never, occasionally, once/year, at least twice/year), and periodontitis (severe/moderate periodontitis versus no/mild periodontitis). In addition, the number of teeth with Grade II or III mobility and the type and position of loose teeth in the dental arches (categorized into 5 classes: maxillary anterior teeth, maxillary posterior teeth, mandibular anterior teeth, mandibular posterior teeth, no loose teeth) were entered into the model of self-perceived tooth mobility. Data are presented as the adjusted odds ratio (OR) and 95% confidence intervals (CI). The Hosmer and Lemeshow test was used to quantify the model fit and *p* < 0.05 was considered statistically significant.

## 3. Results

Table 1 provides the agreement between self-perception and clinical examination. Of the study participants, 50% had a correct perception of their gingival health status (gum bleeding), 52% were aware of suffering or not from bad breath (oral malodor), and only 19% perceived having tooth mobility (loose teeth). 

The basic characteristics of the study population are described in Table 2. The majority of the study subjects were between 40 and 65 years of age, were females, and had a low and middle education level. Over three-quarters were diagnosed as having moderate or severe periodontitis according to the CDC/AAP definition, and almost one-fourth of subjects reported smoking daily. As shown in the bivariate analysis, there was a statistically significant association of all outcome variables with age and education level. A statistically significant association was also verified for gender with self-awareness of gingival bleeding (*p* = 0.001) and oral malodor (*p* < 0.001), for smoking status with self-awareness of oral malodor (*p* = 0.003) and self-reported tooth mobility (*p* < 0.001), and for periodontitis with self-awareness of oral malodor and self-reported tooth mobility (both *p* < 0.001).

Table 3, Table 4 and Table 5 present the results of logistic regression analyses. The self-awareness model of gingival bleeding (Table 3) showed that females were more objective than males in perceiving their own gingival conditions (OR = 1.70, *p* = 0.001), while heavy smokers (OR = 0.62, *p* = 0.045) and individuals in the age group ≥60 years compared to individuals in the younger age group (OR = 0.48, *p* = 0.002) were less likely to report them correctly. FMBS percentages higher than 50% were significantly associated with correct perception of gum bleeding (OR = 2.11 *p* = 0.001), but subjects who reported brushing their teeth twice a day were less objective than those who brushed them less frequently (OR = 0.51, *p* = 0.006).

The oral malodor model (Table 4) indicated that age (older individuals versus younger, OR = 0.55, *p* = 0.007), periodontitis (severe and moderate periodontitis versus no/mild periodontitis, OR = 0.51, *p* = 0.001), and smoking status (heavy smokers versus non-smokers, OR = 0.52, *p* = 0.007) were significant negative predictors of the self-awareness of mouth odor. In contrast, female gender (OR = 1.56, *p* = 0.006) and high compliance to professional oral hygiene sessions (at least twice per year versus never, OR = 1.92, *p* = 0.009) increased the odds of being objective in recognizing their own oral odor.

As regards the tooth mobility model (Table 5), heavy smoking (OR = 2.21, *p* = 0.031) and severe periodontitis (OR = 2.09, *p* = 0.008) increased the odds of perceiving tooth mobility, while age and gender did not. The perception of tooth mobility was also significantly associated with the number of teeth with Grade II and III mobility (OR = 1.45, *p* = 0.001) and tooth position. In particular, the association was stronger for anterior teeth in the mandibular (OR = 10.34, *p* < 0.001) and maxillary arch (OR = 6.38, *p* < 0.001). 

## 4. Discussion

A patient aware of his/her oral conditions is more likely to seek clinical dental care and to adhere more firmly to it [40]. Patient’s behavior is affected not only by the real needs for treatment, but also by oral health-related perceptions and cultural beliefs [41]. 

Few data are available in the current literature on self-awareness of oral health status, and most information relies on selected groups [9,42,43]. The present study included a representative sample of dentate adults living in a city in Northern Italy who answered questions for periodontal symptoms in terms of gingival bleeding, oral malodor, and tooth mobility and underwent a full-mouth clinical examination. 

Gingival bleeding is an early clinical sign of periodontal disease and a key risk marker for existing periodontal inflammation [4] and for the development and progression of periodontitis [44]. The absence of gingival bleeding has been reported as an indicator of periodontal stability [45]. 

We used a gingival inflammation threshold of 10% FMBS, as reported by the current case definition of gingivitis, and a threshold >30% for generalized gingival inflammation [39]. Previous investigations demonstrated that self-reported bleeding was a symptom with high specificity, but low sensitivity [13,14,46]. 

In the current study, the cut-off point beyond which patients were more likely to identify themselves as having gingival disease correctly was 50% of FMBS. Such data indicated that patients were aware of bleeding after toothbrushing and may seek professional treatment only when they already suffered from generalized gingival inflammation. Among Swedish and South American adults, self-reported bleeding resulted in sensitivity ranging from 0.42 to 0.51 for a threshold of ≥50% of bleeding sites [9,47]. After dichotomizing as less and more than 40% of bleeding sites, the sensitivity for occasional bleeding was 0.88 in a Scottish sample of adult patients [15]. 

From the sociodemographic and lifestyle variables, gender, age, smoking habit, and frequency of toothbrushing were factors significantly associated with self-awareness of gingival bleeding, while education level was not. Females were 1.7 times more likely to perceive their own gingival condition correctly than men. In agreement with previous reports, they tended to be more objective in their overall oral health self-perception and self-reported gingival bleeding [42,43]. This could be attributed to the fact that females have more interest in their body appearance and exhibit better oral health knowledge and positive dental behavior than males [48,49]. 

Interestingly, adults aged 60 to 75 years were less likely to self-perceive the symptoms of gingival disease correctly compared to young people. Studies have shown that older people in general tend to overestimate their own oral health compared to young and middle-aged adults, despite the age-associated decline in health status [47,50,51,52]. The factors that affect self-reported oral health are somewhat unclear, but it has been suggested that subjective reactions to oral conditions strongly influence self-perceived oral health and that this is likely more pronounced in the younger age groups [52]. It has been also reported in the elderly that perception of treatment need decreases with increasing age [21]. This could result from older adults’ adaptation process or the recognition of the deterioration of health conditions as normal in aging.

Heavy smoking (more than 10 cigarettes per day) was negatively related to self-awareness of gingival status. Since smoking attenuates the association between plaque and bleeding on probing in a dose-dependent manner, we considered in the logistic model the number of cigarettes smoked per day [53]. Thus, heavy smokers might not recognize the early symptoms of periodontal disease because tobacco consumption reduces the likelihood for gingival bleeding, both subjectively and clinically assessed [53,54,55]. This may be due to decreased vascular density and angiogenesis in the swollen gingiva of smokers compared to non-smokers that mask clinical signs of inflammation [56]. 

Furthermore, although smokers have shown worse self-perceived oral health than non-smokers they are more likely to attend the dentist when the disease is in a more advanced stage [57]. 

A surprising finding was the negative association between toothbrushing twice a day and self-awareness of gingival bleeding. It is important to recognize the potential social desirability bias in reporting the frequency of toothbrushing [58]. It is possible that respondents over-reported behaviors that they believed more desirable for the purpose of the study. A recent Italian survey reported low rates of regular toothbrushing with a percentage as high as 75% of people reporting brushing only once a day [59]. Additionally, the frequency of toothbrushing does not reflect the quality of plaque control. In a companion paper, approximately 87% of the individuals had poor oral hygiene and more than 25% of sites harboring bacterial plaque [3].

The patterns of explanatory variables were similar for both models of self-awareness of gingival bleeding and oral malodor. In the present study, we considered only subjects who perceived their oral malodor themselves and not those informed by others. Self-estimation of bad breath has been demonstrated to be largely unreliable and to have low to moderate sensitivity and specificity [15,16,18]. This is because it is difficult for individuals to assess their own breath due to the psychological and social implications [60]. 

We also found that female gender, age, and tobacco use were significantly related to the self-awareness of halitosis. Differences in perception of overall self-image in females and older people may be indirectly responsible for these findings [61]. A survey among a representative sample of the Dutch population showed that participants aged 60 years and older judged their oral odor as fresher than younger people and that women were more worried than men about their self-perceived mouth odor when meeting another person [62]. 

Smoking has been defined as an independent extrinsic cause of oral halitosis [63]. Correlations between smoking and self-perceived malodor are consistently found in the literature, especially when data are based on questionnaires [64], while no associations between smoking and organoleptic measurements have been reported in some studies [65,66]. In the present study, heavy smoking was negatively associated with the concordance between mouth odor perception and OLT evaluation. It is possible that an unpleasant intraoral taste can lead to poor subjective perceptions about bad breath [67].

Dental visit frequency was the only other significant factor influencing self-awareness of halitosis in the present population. People who have regular dental check-ups may have had the opportunity to receive information on halitosis from dental health professionals that would make them aware of their oral malodor [13]. 

As far as we know, no study investigated factors related to self-perceived tooth mobility. We used Miller’s four-grade index to score mobility at the tooth level [36]. The lack of an index on a dentition-wide basis prevented any comparison between self-perceived and clinically diagnosed dental mobility.

Self-reported presence of tooth mobility was previously found to be a significant predictor of periodontitis with high specificity and low sensitivity across different epidemiological surveys and populations [12,68,69], and its degree was associated with advanced stages of the disease [20,70]. 

Severe periodontitis and smoking more than 10 cigarettes per day emerged as significant predictors of self-perceived tooth mobility in this study. The grade of tooth mobility was positively associated with the severity of periodontal destruction in terms of probing depth and clinical attachment level values and amount of radiographic bone loss [71,72,73]. There is considerable scientific evidence that smoking increases the susceptibility to periodontitis in a dose-dependent manner and is associated with a higher level of periodontal destruction [74]. Heavy smokers presented statistically significantly higher tooth mobility scores than non-smokers or former-smokers, which could be attributed to increased attachment loss and breakdown of alveolar bone [75,76]. 

The number of Degree II and III mobile teeth and the position of mobile teeth in the dental arches were also found to be significantly associated with self-reported mobility in the present study. As expected, the likelihood of self-perceiving tooth mobility increased with the number of involved teeth. Interestingly, patients were more likely to report dental mobility when single-rooted teeth were involved and mostly when they were located at the lower arch. It has been observed that single-rooted teeth exhibit a higher degree of mobility compared to molar teeth [77]. This may be explained by the fact that anterior teeth have a conical single root and lower root surface area with connective tissue attachment compared to posterior teeth in both arches [77]. Furthermore, the diameter and root surface area of the mandibular anterior teeth are lower than those of the maxillary anterior teeth. 

It is important to underline that the accuracy and validity of data from a questionnaire-based survey are heavily influenced by population characteristics, such as cultural background, awareness, socio-economic status, and dental care utilization [78]. Therefore, the present findings could not be generalizable to other populations. Another aspect to be considered is that the agreement between questionnaire and clinical examination depends on the threshold used to define gingival disease and oral malodor. Although OLT is still regarded as the reference standard for clinical oral malodor diagnosis, it has a certain degree of subjectivity and should be complemented with instrumental analysis of breath. 

## 5. Conclusions

Most studies used oral health self-reported responses to interview or a questionnaire to construct predictive models for periodontitis in population-based surveys, while few focused on concordance between self-reports and clinical evaluation and on factors associated with these. We found that only 50% of the subjects were aware of their actual gingival condition and oral malodor. This questions the validity of self-reported measures for surveillance of periodontitis. Females, young subjects, and non-smokers or light smokers were more likely to be objective in scoring their periodontal status. These findings reflected a low level of self-awareness that may influence oral care-seeking behavior. Subjects might be unconcerned about their periodontal health condition or lack enough knowledge to be aware of it. This points to the need for planning strategies to improve education and knowledge about periodontal health, which, enhancing self-perception of symptoms, could help everyone to seek treatment in the initial stage of the disease. 

## Figures and Tables

**Table 1:** Agreement between self-perceived and clinically diagnosed gingival bleeding and oral malodor. FMBS, full-mouth bleeding score; OLT, organoleptic test.

Clinical Evaluation	Self-Perceived Gum Bleeding		Total
	**Yes**	No	
Yes (FMBS ≥ 10%)	310	356	666
No (FMBS < 10%)	10	60	70
**Total**	320	416	736
**Organoleptic Evaluation**	**Self-Perceived Oral Malodor**		**Total**
	**Yes**	No	
Yes (OLT 2–5)	129	278	407
No (OLT 0–1)	279	50	329
**Total**	408	328	736

**Table 2:** Characteristics of subjects according to self-awareness of gingival bleeding and oral malodor and self-perception of tooth mobility.

Variables	Self-Awareness of Gingival Bleeding			Self-Awareness of Oral Malodor			Self-Perception of Tooth Mobility			Total
	Yes	No		Yes	No		Yes	No		
	No. (%)	No. (%)	*p* Value	No. (%)	No. (%)	*p* Value	No. (%)	No. (%)	*p*-Value	
**Gender**			0.001			<0.001			0.370	
Female	238 (55.2)	193 (44.8)		250 (58.0)	181 (42.0)		79 (18.3)	352 (81.7)		431 (58.6)
Male	132 (43.3)	173 (56.7)		133 (43.6)	172 (56.4)		64 (21.0)	241 (21.0)		305 (41.4)
**Age group (years)**			0.004			<0.001			<0.001	
<40	111 (56.6)	85 (43.4)		122 (62.2)	74 (37.8)		12 (6.1)	184 (93.9)		196 (26.6)
40–59	182 (52.1)	167 (47.9)		182 (52.1)	167 (47.9)		81 (23.2)	268 (76.8)		349 (47.4)
60–75	77 (40.3)	114 (59.7)		79 (41.4)	112 (58.6)		50 (26.2)	141 (73.8)		191 (26.0)
**Education**			0.075			0.014			0.001	
Low	139 (45.6)	166 (54.4)		140 (45.9)	165 (54.1)		78 (25.6)	227 (74.4)		305 (41.4)
Middle	156 (54.9)	128 (45.1)		156 (54.9)	128 (45.1)		52 (18.3)	232 (81.7)		284 (38.6)
High	75 (51.0)	72 (49.0)		87 (59.2)	60 (40.8)		13 (8.8)	134 (91.2)		147 (20.0)
**Smoking status**			0.109			0.003			<0.001	
Non-smoker	295 (52.4)	268 (47.6)		307 (54.5)	256 (45.5)		91 (16.2)	472 (83.8)		563 (76.5)
Light smoker (≤10 cigarettes/day)	34 (44.7)	42 (55.3)		41 (53.9)	35 (46.1)		18 (23.7)	58 (76.3)		76 (10.3)
Heavy smoker (>10 cigarettes/day)	41 (42.3)	56 (57.7)		35 (36.1)	62 (63.9)		34 (35.1)	63 (64.9)		97 (13.2)
**Periodontitis**			0.338			<0.001			<0.001	
No	79 (47.0)	89 (53.0)		115 (68.5)	53 (31.5)		8 (4.8)	160 (95.2)		168 (22.8)
Yes	291 (51.2)	277 (48.8)		268 (47.2)	300 (52.8)		135 (23.8)	433 (76.2)		568 (77.2)
Total	370 (50.3)	366 (49.7)		383 (52.0)	353 (48.0)		143 (19.4)	593 (80.6)		736 (100)

**Table 3:** Multivariate logistic model, considering self-awareness of gingival bleeding as the outcome variable (Hosmer and Lemeshow χ^2^ = 11.934, df = 8, *p* = 0.154).

Variables	Adjusted Effect		
	OR	95% CI	*p*-Value
**Gender**			
*Male*	1.00		
*Female*	1.70	(1.23, 2.35)	0.001
**Age (years)**			
*<40*	1.00		
*40–59*	0.82	(0.56, 1.20)	0.309
*60–75*	0.48	(0.30, 0.76)	0.002
**Education**			
*Low*	1.00		
*Middle*	1.42	(0.99, 2.03)	0.058
*High*	1.25	(0.81, 1.93)	0.307
**Smoking status**			
*Non-smoker*	1.00		
*Light smoke*	0.70	(0.42, 1.16)	0.164
*Heavy smoker*	0.62	(0.38, 0.99)	0.045
**Toothbrushing frequency**			
≤*once/day*	1.00		
*twice/day*	0.51	(0.32, 0.83)	0.006
*≥three times/day*	0.70	(0.43, 1.14)	0.154
**FMBS (%)**			
*0–29*	1.00		
*30–49*	0.95	(0.65, 1.39)	0.794
*50–75*	2.11	(1.37, 3.24)	0.001
*>75*	3.60	(2.16, 6.00)	<0.001

**Table 4:** Multivariate logistic model, considering self-awareness of oral malodor as the outcome variable (Hosmer and Lemeshow χ^2^ = 8.652, df = 8, *p* = 0.372).

Variables	Adjusted Effect		
	OR	95% CI	*p*-Value
**Gender**			
*Male*	1.00		
*Female*	1.56	(1.14, 2.12)	0.006
**Age (years)**			
*<40*	1.00		
*40–59*	0.81	(0.55, 1.20)	0.297
*60–75*	0.55	(0.34, 0.84)	0.007
**Education**			
*Low*	1.00		
*Middle*	1.17	(0.82, 1.67)	0.397
*High*	1.24	(0.80, 1.94)	0.334
**Smoking status**			
*Non-smoker*	1.00		
*Light smoker*	0.90	(0.54, 1.48)	0.670
*Heavy smoker*	0.52	(0.32, 0.84)	0.007
**Periodontitis**			
*No*	1.00		
*Yes*	0.51	(0.35, 0.76)	0.001
**Professional scaling frequency**			
*Never*	1.00		
*Occasionally*	1.26	(0.79, 1.99)	0.336
*Once/year*	1.06	(0.73, 1.54)	0.780
*At least twice/year*	1.92	(1.18, 3.15)	0.009

**Table 5:** Multivariate logistic model, considering self-perception of tooth mobility as the outcome variable (Hosmer and Lemeshow χ^2^ = 8.806, df = 8, *p* = 0.359).

Variables	Adjusted Effect		
	OR	95% CI	*p*-Value
**Gender**			
*Male*	1.00		
*Female*	1.05	(0.65, 1.70)	0.844
**Age (years)**			
*<40*	1.00		
*40–59*	1.87	(0.86, 4.04)	0.113
*60–75*	1.75	(0.74, 4.11)	0.201
**Education**			
*Low*	1.00		
*Middle*	0.99	(0.59, 1.68)	0.982
*High*	0.48	(0.22, 1.04)	0.064
**Smoking status**			
*Non-smoker*	1.00		
*Light smoker*	1.38	(0.71, 2.69)	0.338
*Heavy smoker*	2.21	(1.07, 4.54)	0.031
**Severe periodontitis**			
*No*	1.00		
*Yes*	2.09	(1.21, 3.61)	0.008
**Number of teeth with mobility of Grade II or III**	1.45	(1.22, 1.72)	0.001
**Type of loose teeth and location in the arch**			
*No mobile teeth*	1.00		
*Maxillary anterior teeth*	6.38	(3.12, 13.03)	<0.001
*Maxillary posterior teeth*	3.33	(1.47, 7.57)	0.004
*Mandibular anterior teeth*	10.34	(4.99, 16.39)	<0.001
*Mandibular posterior teeth*	4.41	(1.93, 10.09)	0.001
